# Supplementary material for: The impact of perinatal maternal stress on the maternal and infant gut and human milk microbiomes: A scoping review protocol
Source: PLoS One. 2024 Jun 5;19(6):e0304787. doi: 10.1371/journal.pone.0304787 (PMC11152305; doi:10.1371/journal.pone.0304787)
Supplement: S1 File — (DOCX) [file pone.0304787.s001.docx]

**Search Strategy Scoping Review – CINAHL extract**

A three-step process was followed as recommended for JBI reviews.

1. An initial limited search of PubMed and CINAHL was undertaken to identify articles on this topic, followed by analysis of all the text words and keywords contained in the titles and abstracts, and of the index terms used to describe these articles. ✓
2. This informed the development of a second search strategy using all identified keywords and index terms across all included databases. Synonyms and interchangeable terms for these keywords were also collected. The keywords and search terms were peer reviewed and approved by a librarian in UCC. ✓
3. The reference list of all identified reports and articles will be searched for additional studies.✓

One single combination search to map these concepts will not yield the broad results required. Therefore, multiple top up searches in the literature was completed within each research objective 1 – 3 . ( Review 3 searches below per each sub question in each database which I have colour co-ordinated CINAHL, Scopus, Pubmed, Psych Info, Web of Science ).

All searches included text words in the title (TI) and abstract (AB), along with the use of Boolean operators AND and OR within the PCC as described in the Protocol (Table 3).✓

For CINAHL Headings exploded subject headings (MHþ) and major subject headings (MM) was used, with Mesh headings as required in PubMed, followed by Psychological Index terms for Psych info. The asterisk operator * was applied to find variant spellings and word endings in text word terms. ✓

Finally, the reference list of all included sources of evidence will be screened for additional studies. There will be an inclusion of the studies if the papers contain at least **one** of the search terms this will be followed by subtopic categorisation relating to the overarching research topic. Information source from the following databases and search engines will be included: CINAHL Complete (EBSO), Psych Info (EBSO) , Pubmed, Web of Science, Scopus. The searches was limited to English studies and human subjects within the years of 2013 to 2023.

|  | **16.03.23 – CINAHL Full text Database. Main concept Search 1:** | |
| --- | --- | --- |
| **Research topic** | **What is known about the** **impact of perinatal stress on the human milk , maternal and infant gut microbiome** | |
| **Sub question A** | 1. **Does perinatal stress alters the maternal or infant gut microbiome ?** | |
| **Search Strategy** | **Key concepts** | **Synonyms/alternative terminology (consider regional variations here also) – combine using OR** |
|  | *Maternal OR mother OR woman OR women OR female OR MH ”Mothers+”*  AND | |
|  | Prenatal OR antenatal OR antepartum OR Pre-birth OR Perinatal OR Prepartum OR Peri-partum OR Pregnancy OR preg* OR Gestation OR Postnatal OR Postpartum | |
|  | AND  Stress OR anxiety OR “mental health” OR “emotional health” OR “psychosocial stress” OR “mood disorders” OR MH “Stress Physiological OR MH “Stress psychologic” OR MH “Anxiety+” Or MM “Mental Health”  AND | |
|  | Microbiome OR Microbiota OR “Gut Microbiome” OR “Gut microbiota” OR “intestinal microbiota” OR “gastrointestinal microbiome” OR "gastrointestinal microbiota" OR flora OR MM “Gut microbiota”. | |
|  | **16.03.23 – CINAHL Full text Database: Top up search 2** | |
| **Research topic** | **What is known about the impact of maternal stress on the human milk, maternal and infant gut microbiome.** | |
| **Sub question B :** | **B.**  **Does perinatal maternal stress impact on the human milk microbiome?** | |
| **Search Strategy** | **Key concepts** | **Synonyms/alternative terminology (consider regional variations here also) – combine using OR** |
|  | *Maternal OR mother OR woman OR women OR female OR MH ”Mothers+”*  AND | |
|  | Prenatal OR antenatal OR antepartum OR Pre-birth OR Perinatal OR Prepartum OR Peri-partum OR Pregnancy OR preg* OR Gestation OR Postnatal OR Postpartum | |
|  | AND  Stress OR anxiety OR “mental health” OR “emotional health” OR “psychosocial stress” OR “mood disorders” OR MH “Stress Physiological OR MH “Stress psychologic” OR MH “Anxiety+” Or MM “Mental Health” | |
|  | AND  "human milk microbiome" or "milk microbiome" or "HMM" or “breastmilk microbiome” OR "human milk composition" OR MH” Human Milk | |

|  | **16.03.23 – CINAHL Full text Database. Top up Search 3:** | |
| --- | --- | --- |
| **Research topic** | **What is known about the impact of perinatal stress on the human milk and infant gut microbiome and its association with child overweight and obesity?** | |
| **Sub question D:** | **D.** **Is there any evidence to identify the relationship between maternal stress, an altered gut or milk microbiome** | |
| **Search Strategy**  AND | **Key concepts** | **Synonyms/alternative terminology (consider regional variations here also) – combine using OR** |
|  | *Maternal OR mother OR woman OR women OR female OR MH ”Mothers* | |
|  | Prenatal OR antenatal OR antepartum OR Pre-birth OR Perinatal OR Prepartum OR Peri-partum OR Pregnancy OR preg* OR Gestation OR Postnatal OR Postpartum OR MM “Perinatal Period”. | |
|  | AND  Stress OR anxiety OR “mental health” OR “emotional health” OR “psychosocial stress” OR “mood disorders” OR MH “Stress Physiological OR MH “Stress psychologic” OR MH “Anxiety+” Or MM “Mental Healt  AND  h | |
|  | Microbiome OR Microbiota OR “Gut Microbiome” OR “Gut microbiota” OR “intestinal microbiota” OR “gastrointestinal microbiome” OR "gastrointestinal microbiota" OR flora OR MM “Gut microbiota | |
|  | "human milk microbiome" or "milk microbiome" or "HMM" or “breastmilk microbiome” OR "human milk composition" OR MH” Human Milk | |
