# Supplementary material for: The impact of perinatal maternal stress on the maternal and infant gut and human milk microbiomes: A scoping review protocol
Source: PLoS One. 2024 Jun 5;19(6):e0304787. doi: 10.1371/journal.pone.0304787 (PMC11152305; doi:10.1371/journal.pone.0304787)
Supplement: S2 File — (DOCX) [file pone.0304787.s002.docx]

**DATA EXTRACTION TEMPLATE:**

**General information**

**Study ID**

**Title**

Title of paper / abstract / report that data are extracted from

**Lead author contact details**

**Country in which the study conducted**

1. United States
2. UK
3. Canada
4. Australia
5. Europe
6. Other

**Notes**

**Characteristics of included studies**

**Methods**

**Aim of study**

**Study design**

1. Randomised controlled trial
2. Non-randomised experimental study
3. Cohort study
4. Cross sectional study
5. Other

**Start date**

**End date**

**Study funding sources**

**Possible conflicts of interest for study authors**

**Participants**

**Population description**

Mothers and infants , mothers only or infants only

**Variables/ Concepts in the study**

1. Maternal stress and breast milk microbiome
2. Maternal stress and maternal gut microbiome
3. Maternal stress and infant gut microbiome
4. Maternal stress and breast milk microbiome, maternal gut microbiome, infant gut microbiome and child obesity

**Co- variates included**

**Adjusting for Potential confounders**

1. Yes
2. No

**Inclusion criteria**

**Exclusion criteria**

**Method of recruitment of participants**

1. Phone
2. Mail
3. Clinic patients
4. Voluntary
5. Other

**Total number of participants**

**Infants**

**Mothers**

**Baseline Population variable**

|  | **Yes/no** | **Age** | **Measured by** |
| --- | --- | --- | --- |
| **Infant Gut Microbiome** |  |  |  |

**Baseline Population variable contd**

|  | **Yes/no** | **Milk microbiome** | **Gut microbiome** | **Measured by** |
| --- | --- | --- | --- | --- |
| **Pregnant mother** |  |  |  |  |
| **Post natal mother** |  |  |  |  |

**Psychosocial stress measurement**

**Table preview**

|  | **Name** | **Reliability tests** | **What this data extrapolated from other data. Yes/no** | **Where other measurements of stress used such as blood sampling** |
| --- | --- | --- | --- | --- |
| **Self assessment tool used** |  |  |  |  |

**Microbiome Analysis**

The most commonly seen phyla in a typical gut microbiome include Bacteroidetes, Firmicutes, Actinobacteria, Proteobacteria. Are any significant results presented at phylum level? If so please note

Genus level is a further classification from phylum can be noted as Staphylococcus, Streptococcus, Lactobacillus. If discussed as outcome please note.?

Diversity and richness can be measured using Shannon index or Simpsons index.

Summary of outcome

|  | **Measured** | **Statistical analysis** | **SD/ P values** | **Timeline** | **Summary** |
| --- | --- | --- | --- | --- | --- |
| **Phylum level analysis** |  |  |  |  |  |
| **Genus level analysid** |  |  |  |  |  |
| **Microbial diversity** |  |  |  |  |  |

**Maternal stress analysis**

|  | **Mean** | **SD** | **Timeline** | **Summary** |
| --- | --- | --- | --- | --- |
| **Psychosocial stress and microbiome** |  |  |  |  |

**Limitations**

**Final Discussion points or Summary to answer the objective/ review question :**
