# Supplementary material for: The impact of perinatal maternal stress on the maternal and infant gut and human milk microbiomes: A scoping review protocol
Source: PLoS One. 2024 Jun 5;19(6):e0304787. doi: 10.1371/journal.pone.0304787 (PMC11152305; doi:10.1371/journal.pone.0304787)
Supplement: S1 Table — (DOCX) [file pone.0304787.s005.docx]

|  | Key words for Literature search strategy | | | | |
| --- | --- | --- | --- | --- | --- |
| Searches to match multiple Concepts | | **Population AND** | **Concept 1 AND** | **Concept 2 AND** | **Context** |
| 1. Impact of Maternal stress on gut microbiome(s) | | Maternal OR mother OR woman OR women OR female | Stress OR anxiety OR “mental health” OR “emotional health” OR “psychosocial stress” OR “mood disorders” | Microbiome OR Microbiota OR “Gut Microbiome” OR “Gut microbiota” OR “intestinal microbiota” OR “gastrointestinal microbiome” OR "gastrointestinal microbiota" OR flora | Prenatal OR antenatal OR antepartum OR Pre-birth OR Perinatal OR Prepartum OR Peri-partum OR Pregnancy OR preg* OR Gestation OR Postnatal OR Postpartum |
| 1. Maternal stress and human milk microbiome | | Maternal OR mother OR woman OR women OR female | Stress OR anxiety OR “mental health” OR “emotional health” OR “psychosocial stress” OR “mood disorders | "human milk microbiome" or "milk microbiome" or "HMM" or “breastmilk microbiome” OR "human milk composition" | Prenatal OR antenatal OR antepartum OR Pre-birth OR Perinatal OR Prepartum OR Peri-partum OR Pregnancy OR preg* OR Gestation OR Postnatal OR Postpartum |
| 1. Interplay of all concepts   Maternal stress, maternal and/or infant gut microbiome, and human milk microbiome | | Maternal OR mother OR woman OR women OR female | Stress OR anxiety OR “mental health” OR “emotional health” OR “psychosocial stress” OR “mood disorders | Microbiome OR Microbiota OR “Gut Microbiome” OR “Gut microbiota” OR “intestinal microbiota” OR “gastrointestinal microbiome” OR "gastrointestinal microbiota" OR flora | Prenatal OR antenatal OR antepartum OR Pre-birth OR Perinatal OR Prepartum OR Peri-partum OR Pregnancy OR preg* OR Gestation OR Postnatal OR Postpartum |
|  |  |  |  | "human milk microbiome" or "milk microbiome" or "HMM" or “breastmilk microbiome” OR "human milk composition |  |
