# Supplementary material for: The impact of perinatal maternal stress on the maternal and infant gut and human milk microbiomes: A scoping review protocol
Source: PLoS One. 2024 Jun 5;19(6):e0304787. doi: 10.1371/journal.pone.0304787 (PMC11152305; doi:10.1371/journal.pone.0304787)
Supplement: Attachment — Submitted filename: Response to reviewers comments .docx [file pone.0304787.s006.docx]

**The impact of perinatal maternal stress on the maternal and infant gut and human milk microbiomes: A scoping review protocol**

The authors would like to thank the reviewers of PLOS one for their valuable feedback regarding the manuscript. As requested, below are our point-by-point responses to the recommendations made by reviewer 1 & 2 and the page within the revised manuscript where the corresponding changes appear highlighted in track changes.

| **Comment** | **Reply** |
| --- | --- |
| **Reviewer 1** |  |
| Kindly correct the numbering of "The S File 2. Data extraction template for Covidence". Upon download, it shows as S File 3. Data extraction template for Covidence. | Amended now to correct naming of S File 2. Data extraction template for Covidence. |
| For the data extraction sheet, you might consider specifying the biomarker tested for studies in which blood sampling was carried out and separately reporting Shannon α, β as measures of diversity. | Added to Data extraction table “Specify if other biomarkers are used to measure stress, example, cortisol”  With regards to reporting diversity I have added in table to note if alpha or beta diversity is measured. I have added a new column which allows separate reporting of what statistical method is used as there are various ones within these type of studies. |
| **Reviewer 2:** |  |
| In view of the limited number of studies that focus on the effect of maternal perinatal stress on gut microbiome, I recommend that the author may consider including data sources in addition to the peer reviewed articles and systematic reviews, such as book chapters and guideline. Although the authors mentioned the challenges they faced during data extraction from those data sources, but looking into these sources may avoid missing potentially relevant data | Preliminary searches found no guidelines or systematic reviews. The aim of the review was to only include peer reviewed empirical research and while book chapters may go through an editorial process, the content generally does not go through the same peer review process as journals. Therefore, a decision may made not to include book chapters. Grey literature lacks rigorous peer review along with risk of bias and reliability concerns (Adams et al. 2017). Furthermore, in the absence of ample published research, excluding grey literature ensures higher quality, minimizes bias, and maintains objectivity in this review process. |
| In order to improve the search results, the authors may consider adding some key words such as metagenomics, biofilms, dysbiosis and microflora | Amended: These terms will be added to the microbiome synonyms to attempt to widen the search further. |
| Finally, I recommend adding a few sentences to the manuscript discussing how would this scoping review help in improving the health of pregnant women and newborn. | Amended in response to reviewer: End of introduction pg. 4  This review highlights the importance of perinatal stress for maternal and infant well-being. It emphasizes the necessity for clinician intervention and highlights research gaps. By tackling perinatal stress, immediate risks such as low birth weight and preterm delivery could be reduced, while also fostering stronger mother-child bonding (Littleton et al., 2010; McCarthy et al., 2021; Van den Bergh et al., 2005). Furthermore, addressing maternal stress may mitigate negative behaviours like smoking and alcohol consumption thus further improving lifestyle (Matvienko-Sikar et al., 2021; Westerneng et al., 2017). |

Please see below for response to journal requirements.

**Journal Requirements:**

| **Comment** | Response |
| --- | --- |
| 1. Please ensure that your manuscript meets PLOS ONE's style requirements, including those for file naming. | All manuscript checked re formatting style requirements |
| 2. Complete Data Availability Statement in the submission form, | "All data are in the manuscript and/or supporting information files" as Data Availability Statement. |
| 3. We note that you have referenced (unpublished) on page 7, which has currently not yet been accepted for publication. Please remove this from your References and amend this to state in the body of your manuscript: (ie “Bewick et al. [Unpublished]”) as detailed online in our guide for authors | Unable to locate the reference you are referring to. The only reference on page 7 is "Adams RJ, Smart P, Huff AS. Shades of Grey: Guidelines for Working with the Grey Literature in Systematic Reviews for Management and Organizational Studies. International Journal of Management Reviews. 2017 Oct;19(4):432–54." which is published. |
| 4. Please upload a copy of Figure 1, to which you refer in your text on page 4. If the figure is no longer to be included as part of the submission, please remove all reference to it within the text." | Regarding Figure 1 - this was uploaded as Supporting information S.Fig 1. I previously was told to remove images from the manuscript. Rechecking the formatting guideline it states *“Figures should be uploaded separately as individual files”* therefore I have left the Figure 1 as supporting information as before labelled as S.Fig 1. |
| 5. We notice that your supplementary [S Fig 1 and S Fig 2 ] are included in the manuscript file. Please remove them and upload them with the file type 'Supporting Information'. Please ensure that each Supporting Information file has a legend listed in the manuscript after the references list." | The only things submitted within this manuscript was Table 1 - 3. There was no S Fig  1or S Fig 2 within text so I presume this is an error? There was an S Figure 1 cited in text as Figure 1 Concept Map (pg. 5) only which was added as supporting information. |
